# Issue Information

**DOI:** 10.1002/ece3.340

**Published:** 2012-07-06

## Aims and Scope

*Ecology and Evolution* is the peer-reviewed journal for rapid dissemination of research in all areas of ecology, evolution and conservation science. The journal gives priority to quality research reports, theoretical or empirical, that develop our understanding of organisms and their diversity, interactions between them, and the natural environment.

*Ecology and Evolution* gives prompt and equal consideration to papers reporting theoretical, experimental, applied and descriptive work in terrestrial and aquatic environments. The journal will consider submissions across taxa in areas including but not limited to micro and macro ecological and evolutionary processes, characteristics of and interactions between individuals, populations, communities and the environment, physiological responses to environmental change, population genetics and phylogenetics, relatedness and kin selection, life histories, systematics and taxonomy, conservation genetics, extinction, speciation, adaption, behaviour, biodiversity, species abundance, macroecology, population and ecosystem dynamics, and conservation policy.

*Ecology and Evolution* features original research articles, reviews, editorials, and hypotheses. Original research papers must report well-conducted research with conclusions supported by the data presented in the paper.

*Ecology and Evolution* publishes papers submitted directly to the journal and those referred from a select group of prestigious journals published by Wiley-Blackwell. *Ecology and Evolution* is a Wiley Open Access journal, one of a new series of peer-reviewed titles publishing quality research with speed and efficiency. For further information visit the Wiley Open Access website at http://www.wileyopenaccess.com

## Open Access and Copyright

All articles published by *Ecology and Evolution* are fully open access: immediately freely available to read, download and share. All *Ecology and Evolution* articles are published under the terms of the Creative Commons Attribution Non Commercial License which permits use, distribution and reproduction in any medium, provided the original work is properly cited and is not used for commercial purposes.

Copyright on any research article in a journal published by *Ecology and Evolution* is retained by the author(s). Authors grant Wiley a license to publish the article and identify itself as the original publisher. Authors also grant any third party the right to use the article freely as long as its integrity is maintained and its original authors, citation details and publisher are identified.

## Use by Commercial “For-Profit” Organisations

Use of Wiley Open Access articles for commercial, promotional, or marketing purposes requires further explicit permission from Wiley and will be subject to a fee. Copyright © 2012 Blackwell Publishing Ltd. All rights reserved. Requests to reproduce material from this journal content are being handled through the Rightslink® online service.

- Locate the article you wish to reproduce on Wiley Online Library (http://onlinelibrary.wiley.com)
- Navigate to the abstract page.
- Click on the ‘Request Permissions’ link, under the ‘ARTICLE TOOLS’ menu
- Follow the online instructions and select your requirements from the drop down options and click on ‘quick price’ to get a quote
- Create a RightsLink® account to complete your transaction (and pay, where applicable)
- Read and accept our Terms & Conditions and Download your license
- For any technical queries please contact customercare@copyright.com
- For further information and to view a Rightslink® demo please visit http://www.wiley.com and select Rights& Permissions.

Special requests should be addressed to permissionsuk@wiley.com. Print reprints of Wiley Open Access articles can be purchased from corporatesales@wiley.com.

## Disclaimer

The Publisher and Editors cannot be held responsible for errors or any consequences arising from the use of information contained in this journal; the views and opinions expressed do not necessarily reflect those of the Publisher and Editors, neither does the publication of advertisements constitute any endorsements by the Publisher and Editors of the products advertised.

Wiley Open Access articles posted to repositories or websites are without warranty from Wiley of any kind, either express or implied, including, but not limited to, warranties of merchantability, fitness for a particular purpose, or non-infringement. To the fullest extent permitted by law Wiley disclaims all liability for any loss or damage arising out of, or in connection, with the use of or inability to use the content.

